# Distribution of combined p-values for baseline variables in a consecutive sample of randomized controlled trials

**DOI:** 10.1186/s12874-026-03082-x

**Published:** 2026-11-06

**Authors:** Rebecka Klang, Louise Olsson

**Affiliations:** Centre for Assessment of Medical Technology in Örebro (Camtö), Faculty of Medicine and Health, Örebro University, Örebro, Sweden

**Keywords:** Randomization, Baseline variables, P values, Research integrity, Data integrity, Meta research, Statistical method

## Abstract

**Background:**

Combined p-values derived from summary statistics of baseline variables in randomized controlled trials (RCTs) are expected to follow a uniform distribution. Extreme values indicate similarity or dissimilarity across randomized groups. To date, investigations have been limited to a small number of clinical fields; here, we assess the distribution of combined p-values in a consecutive sample of RCTs.

**Methods:**

Based on a PubMed search between February 1 and 18, 2022, 568 RCTs were included. Baseline variables were analysed using Carlisle’s method, yielding one combined p-value per RCT. A threshold of combined p-values of p ≤ 0.05 or p ≥ 0.95 was applied. Differences in combined p-value distributions between subgroups were assessed using the two-sample Anderson-Darling (AD) test.

**Results:**

26.2% (149/568) of the included RCTs had combined p-values of p ≤ 0.05 or p ≥ 0.95, compared with the 10% expected by chance. Of these, 104 RCTs (18.3%) were found in the upper extreme tail of p ≥ 0.95 and 45 RCTs (7.9%) in the lower extreme tail of p ≤ 0.05. Smaller RCTs (N ≤ 100) had more extreme combined p-values than larger RCTs (N > 100) (AD test p = 0.001). RCTs with > 7 baseline variables had more extreme combined p-values than those with ≤ 7 variables (AD test p = 0.004). A sensitivity analysis showed that 20.6% of RCTs still had extreme combined p-values after controlling for reporting errors related to SE and SD. Another sensitivity analysis showed that some extreme results could be explained by assuming high correlations between baseline variables within an RCT.

**Conclusion:**

A substantially higher proportion of RCTs in a consecutive sample exhibited extreme similarities or dissimilarities in baseline characteristics across groups than expected by chance (26.2% vs 10%), indicating problems with randomisation. Further investigation into the causes and implications of implausible baseline distributions in RCTs is warranted.

**Registered:**

2023-03-20 at https://www.researchweb.org/fourol/project/280487

## Background

Randomized controlled trials (RCTs) are considered the gold standard in clinical research for evaluating interventions. However, estimates suggest that approximately 25% of all RCTs have serious flaws, with causes ranging from honest mistakes to deliberate fraud [1, 2]. The randomization process is crucial, and when properly conducted, any baseline differences between intervention and control groups arise purely by chance. Consequently, some degree of baseline variation between the groups is expected.

In 2012, British anaesthesiologist John B. Carlisle reported that the distribution of baseline variables in RCTs of a particular researcher was highly unlikely to have resulted from proper randomization [3]. An investigation of this researcher led to the retraction of 172 papers [4]. The method was subsequently refined and applied to RCTs by another researcher, yielding similar results [5]. Carlisle later applied this approach to 5,087 RCTs published between 2000 and 2015, predominantly in anaesthesiology [6], as well as 526 anaesthesiology RCTs submitted between 2017 and 2020 [2], identifying a higher-than-expected proportion of non-randomness in both samples. Other researchers have applied Carlisle’s method in specific fields, including bone physiology [7–9], reproductive medicine [10], and psychology [11]. The journal Anaesthesia decided in 2016 to start screen all RCTs submitted to the journal with the method [12].

To our knowledge, no study has applied Carlisle’s method to a consecutive sample of published RCTs across healthcare. The aim of this study was therefore to apply the method to a consecutive sample of RCTs in PubMed, without restriction to topic or journal. We hypothesized that (1) more RCTs with extreme baseline distributions, indicating problems with randomization, would be identified than expected by chance alone, and (2) the proportion of RCTs with potential randomization issues would differ from Carlisle’s findings in anaesthesiology from 2000–2015 [6].

## Methods

### Study design

A cross-sectional study of RCTs was conducted to examine baseline variable distribution using Carlisle’s method.

### Literature search and selection of trials

A librarian at the Medical Library at Örebro University searched PubMed on September 15, 2022, for RCTs indexed between February 1 and 18, 2022, using the search string (“Randomized Controlled Trial” [Publication Type:NoExp]) AND (2022/02/01:2022/02/18 [edat]), without applying filters or additional restrictions. The search period was selected to provide a sufficiently large pool of potentially eligible trials, allowing for exclusions during screening while ensuring that the final sample would exceed the required sample size.

The inclusion criteria were RCTs conducted in humans, with full-text available in English, regardless of randomization method. Pilot and feasibility trials, as well as secondary analyses of RCTs, were excluded. Eligibility was assessed by one author (RK) and independently verified by another researcher (see Acknowledgments).

### Data extraction

Metadata for all included RCTs were extracted by one author (RK or LO) and independently verified by another researcher. The extracted metadata included type of intervention, ICD-10 diagnosis, and country of the corresponding author.

Baseline variables were defined as variables measured before exposure to the allocated intervention, e.g., age, height, or laboratory measurements. Means and standard deviations (SD) of all continuous baseline variables from all randomized groups were manually extracted. All standard errors (SE) were converted to SDs. Medians reported with interquartile ranges (IQR) and/or ranges were transformed to means and SDs using Wan et al.’s method [13]. The number of participants in each group, use of stratification in randomization, and the number of decimal places reported were also extracted. Obvious typographical errors were corrected (e.g., a reported weight of 758 kg was corrected to 75.8 kg). When both weight and height were reported, BMI was omitted, as it is derived from these values. All categorical variables were excluded.

Data extraction was conducted by one author (RK) in three stages, with a washout period of several weeks between each stage. In the first stage, means and SDs were extracted. The second stage involved extracting medians with IQR and/or ranges, transforming them to means and SDs, and reviewing previously extracted data. The third stage consisted of a final review of all extracted data.

### Carlisle’s method

A detailed description of Carlisle’s method has been published previously [14]. An example for how to apply the method in practice can be found in Additional file 1. All methodological decisions in this study were based on the published description of Carlisle’s method and the methodological decisions made in Carlisle’s study from 2017 [6]. In brief, extracted baseline variables are used to recompute p-values for differences between the intervention and control groups. This is done using three statistical approaches: Monte Carlo simulations (100,000 iterations), ANOVA, and, for two-group comparisons, Student’s t-test. These approaches yield two or three p-values for each baseline variable. The complement of each p-value (i.e., 1 minus the calculated p-value) is then calculated, and the value closest to 0.5 is selected. Selected p-values across baseline variables within an RCT are then combined using Stouffer’s method [15] to produce a single combined p-value per RCT. If the assumptions of the method are met and RCTs are properly randomized, the resulting combined p-values are expected to follow a uniform distribution. This means that p-values are expected to be evenly distributed across the interval from 0 to 1, with equal proportions falling within intervals of equal length, including those near 0 and 1.

The combined p-value of an RCT thus summarizes the similarity of baseline means between the randomized groups. A combined p-value close to 0 indicates high similarity in baseline means, whereas a combined p-value close to 1 indicates dissimilarity, because the complements of the p-values are used. The threshold used in this study to define overly similar or dissimilar results was arbitrarily set at combined p-values within 0.05 of 0 or 1, i.e., p ≤ 0.05 or p ≥ 0.95.

### Sensitivity and subgroup analyses

Subgroup analyses were conducted to explore factors previously suggested to influence the performance of Carlisle’s method, including the number of participants, the number of baseline variables, the number of decimal places, and the use of stratification in randomization. RCTs with > 100 participants were compared with those with ≤ 100 participants, based on the median sample size as the cutoff. Median values were also used to define subgroups based on the number of variables and decimal places. Differences in the distribution of combined p-values between subgroups were assessed using the two-sample Anderson-Darling (AD) test. P-values from the AD tests were adjusted using the Holm-Bonferroni correction to account for multiple testing.

A sensitivity analysis was conducted to assess the potential impact of errors in the reporting of SDs or SEs, which have previously been identified as a source of extreme combined p-values [6]. To account for the possibility that SDs had been incorrectly reported as SEs, all extracted SDs were multiplied by $\sqrt{{\rm{n}}}$. To account for the reverse situation, where SEs had been incorrectly reported as SDs, all extracted SDs were divided by $\sqrt{{\rm{n}}}$. If the combined p-value of an RCT was no longer “extreme” after either transformation, this result was interpreted as likely reflecting a reporting error in the SD or SE.

To assess the impact of different assumed correlations between baseline variables within an RCT, sensitivity analyses of the primary and subgroup analyses were conducted using Strube’s method [16] instead of Stouffer’s method. Correlation coefficients of 0.2, 0.4, 0.6, and 0.8 were assumed for all pairs of baseline variables within each RCT.

Another sensitivity analysis was conducted to assess whether the use of different reported measures of central tendency affected the resulting distribution. This was done by comparing an analysis based on variables transformed from medians (with IQRs and/or ranges) into means (SDs) with an analysis based on directly reported means. Because some RCTs reported both medians and means across different variables, they could contribute to both analyses, but with different variables. When the same variable was reported as both a median and a mean, the mean value was used.

Further sensitivity analyses were conducted using Fisher’s method [17] instead of Stouffer’s method to generate combined p-values. Another sensitivity analysis used only Monte Carlo simulations to recalculate p-values, excluding ANOVA and Student’s t-test.

### Sample size calculation

A minimum sample size of 255 RCTs was required to detect a difference between an expected proportion of 10% by chance and 15.6% reported by Carlisle [6], assuming a two-sided significance level of 0.05 and 80% power, using a one-sample z-test for proportions. The reference proportion of 15.6% was derived from Carlisle’s predominantly anaesthesiology sample and may not fully reflect the proportion expected in a broader, cross-disciplinary sample of RCTs.

### Software

Data were collected in Excel 2016, and all analyses were carried out in RStudio 2026.07.1 + 147 [18] with R-packages “BSDA” [19], “CarletonStats” [20], “dplyr” [21], “kSamples” [22], “MBESS” [23], “poolr” [24], “qqtest” [25], “readxl” [26] and “writexl” [27].

Stouffer’s method, as implemented in R [18], cannot handle p-values equal to 0 or 1. When p-values are very close to these extremes, R will treat them as exactly 0 or 1. To avoid this, p-values from the Monte Carlo simulation were adjusted slightly: values equal to 0 were set to 1e-16, and values equal to 1 set to 1-1e-16.

The AD test from the “kSamples” package [22] was used. The package provides two versions of the p-value, and statistical significance was assessed using the Version 1 p-value, as recommended by the package documentation when the distribution is continuous. The R code was adapted from code shared by Carlisle [5, 28] and slightly refined. The data and code used for the analyses are provided in Additional files 2 and 3.

## Results

Out of 1,075 articles indexed as RCTs in PubMed during the search period, 568 met the inclusion criteria for this study. Consequently, the number of RCTs included was more than twice the 255 trials estimated to be required by the sample size calculation (flowchart in Additional file 1: Figure S1). More than one third of the trials dealt with pharmacological issues, and almost one fifth originated from the USA (Table 1). The median number of participants per RCT was 102 (IQR 60–270; range 10–48,125).

**Table 1:** Characteristics of included RCTs (n = 568), n (%)

**Diagnosis**	
Endocrine, nutritional, and metabolic diseases	61 (10.7%)
Infectious diseases	53 (9.3%)
Mental and behavioural disorders	53 (9.3%)
Other	401 (70.6%)
**Intervention**	
Pharmacological	208 (36.6%)
Surgery / Procedural	83 (14.6%)
Psychological / Social / Nursing	75 (13.2%)
Other	202 (35.6%)
**Country**^**a**^	
USA	105 (18.5%)
China	72 (12.7%)
Iran	30 (5.3%)
Other	361 (63.6%)
**Number of participants per study**^**b**^	102 (60–270) [10–48,125]
**Number of variables extracted per study**^**b**^	7 (3–13) [1–169]

^a^ If the corresponding author had affiliations in multiple countries, the country of the reported address was used. If no address was reported, the first listed country was used. If multiple corresponding authors were reported, the first listed author was used.

^b^ Median (q1–q3) [min - max]

A total of 6,326 baseline variables were extracted from the included RCTs. In total, 5,270 variables were reported as mean (with SD, or SE transformed to SD) in 498 RCTs, and 1,056 were reported as median (with IQR and/or range) and transformed to mean (SD) from 177 RCTs. In total, 14,530 summary values were extracted for the analyses, due to each variable contributing values from two or more groups. Median number of extracted variables per RCT was 7 (IQR 3–13; range 1–169).

A combined p-value of p ≤ 0.05 or p ≥ 0.95 was observed in 26.2% (149/568) of the included RCTs (Table 2), which is 16.2 percentage points (92 RCTs) above the 10% expected by chance. Specifically, p ≤ 0.05 (indicating high similarity in baseline variables across randomised groups) was observed in 7.9% of RCTs, 2.9 percentage points (16 RCTs) higher than the 5% expected by chance. A p ≥ 0.95 (indicating high dissimilarity in baseline variables), was observed in 18.3% of RCTs, 13.3 percentage points (76 RCTs) higher than the 5% expected by chance. The largest deviation from the expected value was observed in RCTs with a combined p-value of p ≥ 0.999. Only 0.1% would be expected in this group by chance, whereas 9.3% (53 RCTs) fell into this category, 93 times more than expected. A table comparing these results with Carlisle (2017) [6] is provided in Additional file 1: Table S1.

**Table 2:** Extreme combined p-values for included RCTs (n = 568)

Combined p-value	Observed %	Observed number of RCTs	Expected %	Expected number of RCTs
**p** ≤ **0.05**	**7.9**	**45**	**5**	**28.4**
0.05 ≥ p > 0.01	3.5	20	4	22.7
0.01 ≥ p > 0.001	2.8	16	0.9	5.1
p ≤ 0.001	1.6	9	0.1	0.6
**p** ≥ **0.95**	**18.3**	**104**	**5**	**28.4**
0.95 ≤ p < 0.99	5.6	32	4	22.7
0.99 ≤ p < 0.999	3.3	19	0.9	5.1
p ≥ 0.999	9.3	53	0.1	0.6
**p** ≤ **0.05 or** ≥ **0.95**	**26.2**	**149**	**10**	**56.8**

**Fig. 1:**
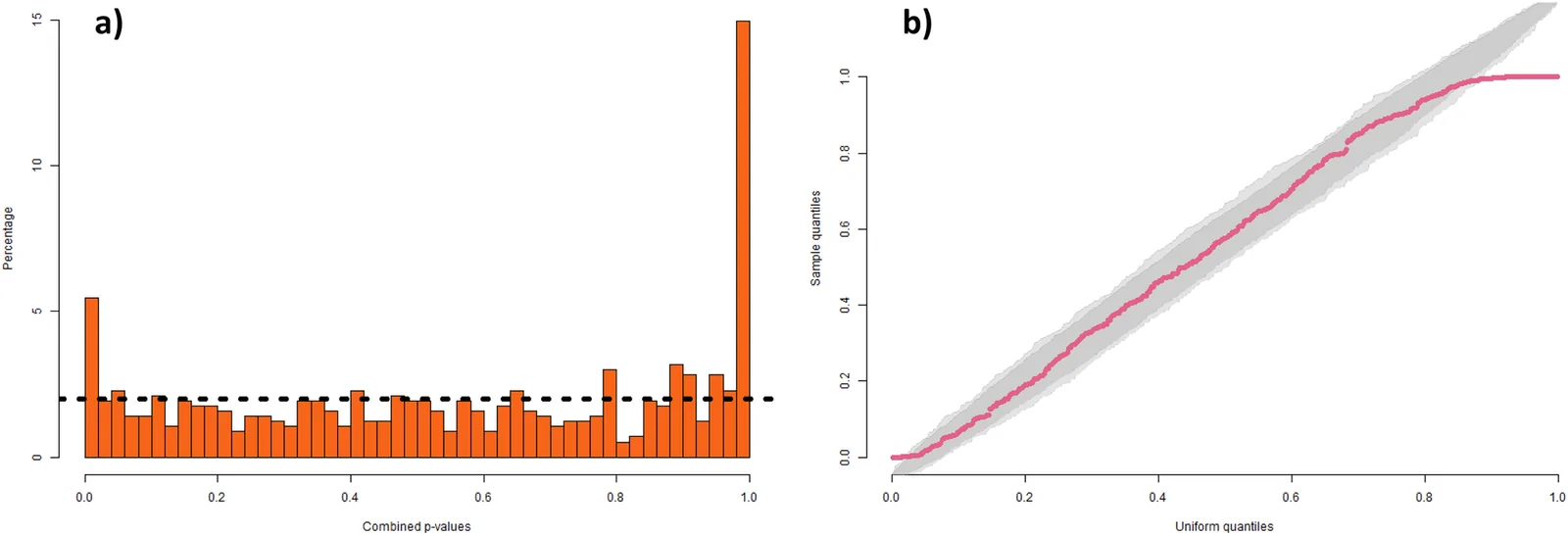
Distribution of combined p-values in the 568 included RCTs, showed by (**a**) a histogram and (**b**) a Q-Q plot with a 99% confidence interval for the expected uniform distribution.

The distribution of combined p-values of p ≤ 0.05 and p ≥ 0.95 is further illustrated in Fig. 1a and b, showing that the number of RCTs with baseline variables deviating from what would be expected by chance is higher in the extreme upper tail. The combined p-values between the extreme tails were approximately uniformly distributed.

### Subgroup analyses

Among RCTs with ≤ 100 participants, 10.8% (30 RCTs) had combined p-values of p ≤ 0.05, compared with 5.2% (15 RCTs) among those with > 100 participants (AD test p = 0.001; Table 3, Additional file 1: Figure S2 and S3). This suggests that smaller RCTs were twice as likely to have highly similar baseline variables across groups than larger trials, which were more closely aligned with the expected proportion in the lower tail.

**Table 3:** Extreme combined p-values for number of participants and baseline variables

		Participants		Baseline variables	
		N > 100 n = 289	N ≤ 100 n = 279	> 7 n = 272	≤ 7 n = 296
**Combined p-value**	**Expected %**	**Observed %**		**Observed %**	
**p** ≤ **0.05**	5	5.2	10.8	11.4	4.7
0.05 ≥ p > 0.01	4	2.8	4.3	4.4	2.7
0.01 ≥ p > 0.001	0.9	2.1	3.6	4.0	1.7
p ≤ 0.001	0.1	0.3	2.9	2.9	0.3
**p** ≥ **0.95**	5	21.1	16.1	23.2	14.2
0.95 ≤ p < 0.99	4	5.9	6.1	6.6	5.1
0.99 ≤ p < 0.999	0.9	4.2	2.5	4.4	2.4
p ≥ 0.999	0.1	11.1	7.5	12.1	6.8
**p** ≤ **0.05 or p** ≥ **0.95**	10	26.3	26.9	34.6	18.9

The proportion of RCTs with combined p-values of p ≥ 0.95, indicating dissimilar baseline characteristics, was 21.1% in larger and 16.1% in smaller trials, corresponding to 4 and 3 times the expected proportion, respectively (Table 3).

Among RCTs with ≤ 7 baseline variables, 4.7% (14 RCTs) had combined p-values of p ≤ 0.05, compared with 11.4% (31 RCTs) among those with > 7 variables (AD test p = 0.004; Table 3 and Additional file 1: Figure S4 and S5). The proportion of RCTs with combined p-values of p ≥ 0.95 was 14.2% (42 RCTs) in trials with ≤ 7 variables and 23.2% (63 RCTs) among those with > 7 variables. Trials with many baseline variables were more likely to show extreme combined p-values, indicating both greater similarity and dissimilarity in baseline characteristics.

No statistically significant differences in the distributions of combined p-values were observed between RCTs reporting 0–1 versus ≥ 2 decimal places (AD test p = 0.07), or between stratified and non-stratified randomization (AD test p = 0.68) (Additional file 1: Table S2).

### Sensitivity analyses

Out of 45 RCTs with combined p ≤ 0.05, 9 (20%) could be explained by SE being reported instead of SD. Out of 104 RCTs with p ≥ 0.95, 23 (22.1%) could be explained by SD being reported instead of SE. Even after accounting for these potential reporting errors, 20.6% (117/568) of the included RCTs had combined p-values of p ≤ 0.05 or p ≥ 0.95 (Table 4).

**Table 4:** Extreme combined p-values after adjustment for potential reporting errors in SD and SE (n = 568)

Combined p-value	Observed %	Observed number of RCTs	Expected %	Expected number of RCTs
**p** ≤ **0.05**	**6.3**	**36**	**5**	**28.4**
0.05 ≥ p > 0.01	3.0	17	4	22.7
0.01 ≥ p > 0.001	2.1	12	0.9	5.1
p ≤ 0.001	1.2	7	0.1	0.6
**p** ≥ **0.95**	**14.3**	**81**	**5**	**28.4**
0.95 ≤ p < 0.99	4.6	26	4	22.7
0.99 ≤ p < 0.999	2.3	13	0.9	5.1
p ≥ 0.999	7.4	42	0.1	0.6
**p** ≤ **0.05 or** ≥ **0.95**	**20.6**	**117**	**10**	**56.8**

As the assumed correlation between baseline variables within an RCT increased, the proportion of RCTs with extreme combined p-values decreased (Table 5). For assumed correlations of 0.4 and above, the proportion of RCTs with combined p-values of p ≤ 0.05 was below the expected 5%, ranging from 1.1 to 3.3%. The proportion of RCTs with combined p-values of p ≥ 0.95 remained above the expected 5% at all correlation levels but decreased as the assumed correlation increased, with the lowest proportion (8.1%) observed at a correlation of 0.8.

**Table 5:** Extreme combined p-values for different assumed correlations among all variables within each RCT (n = 568)

		No correlation	Correlation 0.2	Correlation 0.4	Correlation 0.6	Correlation 0.8
Combined p-value	Expected %	Observed %	Observed %	Observed %	Observed %	Observed %
**p** ≤ **0.05**	**5**	**7.9**	**6.0**	**3.3**	**2.1**	**1.1**
0.05 ≥ p > 0.01	4	3.5	3.5	1.8	1.6	1.1
0.01 ≥ p > 0.001	0.9	2.8	1.9	1.4	0.5	0.0
p ≤ 0.001	0.1	1.6	0.5	0.2	0.0	0.0
**p** ≥ **0.95**	**5**	**18.3**	**16.9**	**13.7**	**10.4**	**8.1**
0.95 ≤ p < 0.99	4	5.6	6.3	5.5	4.0	3.7
0.99 ≤ p < 0.999	0.9	3.3	3.3	3.5	2.8	1.9
p ≥ 0.999	0.1	9.3	7.2	4.8	3.5	2.5
**p** ≤ **0.05 or** ≥ **0.95**	**10**	**26.2**	**22.9**	**17.1**	**12.5**	**9.2**

The impact of assuming different correlations among all variables in the subgroup analyses is shown in Additional file 1: Table S3–S6. In all subgroups, the proportion of RCTs with combined p-values of p ≤ 0.05 fell below the expected 5%. For p ≥ 0.95, the proportions of RCTs with ≤ 100 participants and with > 7 baseline variables approached the expected 5% as the assumed correlation increased. In the remaining subgroups, the proportions ranged from 7.6% to 11.1% at an assumed correlation of 0.8, which consistently resulted in the lowest proportions of combined p-values.

When Fisher’s method was used instead of Stouffer’s method to combine the p-values, the proportion of combined p-values of p ≤ 0.05 was similar to that obtained when Stouffer’s method was used (7.6% vs 7.9%), whereas the proportions of combined p-values of p ≥ 0.95 was lower (14.1% vs 18.3%) (Additional file 1: Table S7). When only Monte Carlo simulations were used to for recalculate the p-values, both end tails were similar to those obtained when also ANOVA and Student’s t-test were used, with 8.5% of combined p-values of p ≤ 0.05 and 19.5% of p ≥ 0.95 (Additional file 1: Table S7).

The proportion of extreme combined p-values differed between the 177 RCTs in which baseline variables were reported as medians and transformed to means and the 498 RCTs in which baseline variables were directly reported as means (Additional file 1: Table S7). Among the RCTs originally reporting baseline variables as medians, 4.5% had combined p-values of p ≤ 0.05, compared with 8.2% among RCTs reporting baseline variables directly as means. For combined p-values of p ≥ 0.95, the corresponding proportions were 24.3% and 15.7%, respectively. No statistically significant difference was, however, observed between their distributions (AD test p = 0.08).

## Discussion

Combined p-values for baseline characteristics in a consecutive sample of 568 RCTs conducted in humans showed that 26.2% had extreme values (p ≤ 0.05 or p ≥ 0.95), 16.2 percentage points higher than the 10% expected by chance. This finding suggests that non-randomness may be present in a substantial proportion of RCTs. Extreme values indicating baseline dissimilarity (p ≥ 0.95) was more than twice as common as those indicating similarity (p ≤ 0.05) (18.3% vs 7.9%).

Our results substantially exceed those reported by Carlisle [6], who found that 15.6% of RCTs had combined p-values of p ≤ 0.05 or p ≥ 0.95. The largest difference was observed for RCTs with combined p-values of p ≥ 0.999, with 9.3% in our study compared with 2.0% reported by Carlisle. One important difference between the studies was the sampling frame. Carlisle’s investigation was based on eight specific journals; six specialist anaesthetic journals and two general medical journals, whereas our sample included all RCTs indexed in PubMed during a defined period. Consequently, our dataset comprises a broader mix of journals with varying impacts and across multiple disciplines, which may provide a more representative sample of RCTs. The prevalence of problematic randomisation may be lower in anaesthesiology RCTs; however, Carlisle did not observe significant differences between anaesthesiology and general medical journals [6]. Differences in time period may also account for the discrepancies, as Carlisle analysed RCTs from 2000 to 2015, whereas we examined RCTs from February 2022. However, our findings are consistent with those of Šimsa, who applied Carlisle’s method to a random sample of RCTs in psychology between 1970 and 2020 [11].

The assumption of a uniform distribution of combined p-values should hold regardless of sample size in RCTs, yet we observed a statistically significant difference between smaller (N ≤ 100) and larger (N > 100) trials, suggesting potential sample-size related differences in baseline patterns. The underlying reasons remain unclear but may reflect differences in randomization quality. Carlisle’s method has been criticized for sometimes relying on few baseline variables, which may allow a single incorrect p-value to disproportionately influence the combined p-value of an RCT [29]. However, we found that RCTs with ≤ 7 baseline variables had fewer extreme combined p-values than those with > 7 variables, suggesting that a smaller number of variables did not increase the proportion of extreme results. One concern regarding the method relate to rounding of reported values [30]. Simulation studies by Bolland et al. have demonstrated that rounding may affect the theoretical distribution of p-values [8, 9], but in our study, no statistically significant difference was observed. Another concern relates to the applicability of the method when stratified randomization is used [29, 30]. Because stratification is intended to achieve greater balance between the randomized groups with respect to selected variables, thereby potentially increasing their similarity, one might expect studies using stratified randomization to have a higher proportion of combined p-values of p ≤ 0.05. In our sample, however, non-stratified RCTs had a higher proportion of p ≤ 0.05, but the difference was not statistically significant. This is consistent with the simulation study by Bolland et al., which found that stratification or other randomization methods do not appear to affect the theoretical distribution of p-values [8].

Our findings remained consistent after accounting for possible reporting errors between SEs and SDs, with 21.5% (32/149) of the extreme combined p-values (p ≤ 0.05 or p ≥ 0.95) potentially attributable to such errors. These errors may reflect typographical mistakes or misunderstanding of the measures, however, we cannot confirm whether the values were truly misreported, as individual RCTs were not examined in detail. Carlisle [6] also demonstrated that errors involving SDs and SEs contributed to extreme combined p-values, which has been highlighted as a limitation of the method [29, 31]. This underscores the importance of accounting for such reporting errors when applying Carlisle’s method. However, the main results of this study remained unchanged. After accounting for the potential SE/SD reporting errors, 20.6% (117/568) of the included RCTs had a combined p-value of p ≤ 0.05 or p ≥ 0.95, which was twice the proportion expected by chance.

Correlations among baseline variables within RCTs has previously been raised as a concern regarding Carlisle’s method [29–32]. It has also been shown that p-values for baseline variables are not uniformly distributed when the variables are correlated [33]. In our sensitivity analyses, we found that strong correlations between all baseline variables within an RCT can substantially affect the results of Carlisle’s method and may lead to false-positive findings. The magnitude of this effect is difficult to estimate, as it is unlikely that all reported baseline variables within each RCT would be correlated as highly as 0.8. These analyses demonstrate that correlations between variables can influence the results and should be considered when applying and interpreting Carlisle’s method.

The sensitivity analysis using Fisher’s method instead of Stouffer’s method produced similar proportions of RCTs with combined p-values of p ≤ 0.05, but a lower proportion with for p ≥ 0.95. This suggests that the findings in the lower tail were relatively robust to the choice of p-value combination method, whereas the excess in the upper tail was more sensitive to this choice.

One difference from Carlisle’s previous study [6] concerns p-values that R treats exactly as 0 or 1. These values were slightly adjusted ( ± 1e–16) and therefore retained in the present analysis, whereas they would have been omitted in Carlisle’s analysis. The impact on the overall conclusion is unclear but should be considered when applying the method.

We did not assess normality or the impact of non-normal data, which is a recognized limitation of Carlisle’s method [30]. Some of the extracted variables were likely skewed (e.g., age in trials of age-associated diseases), although simulation studies suggest that non-normality does not substantially affect the distribution of p-values [8]. In addition, baseline variables were extracted by a single researcher and, although the extraction was conducted and checked in three stages, errors may still have occurred. Sampling variability may affect the classification of RCTs with combined p-values close to the predefined thresholds, as relatively small changes in the observed data could shift RCTs into or out of the extreme tails. The lack of raw data also precluded validation of the findings.

A key strength of the study is the use of a consecutive sample of RCTs covering a broad range of topics and journals, thereby providing a broader overview of the literature. Our findings may offer an important benchmark for future studies in various fields. The conducted subgroup analyses that explore potential heterogeneity also contribute to a better understanding of the method.

The observed excess of RCTs with extreme combined p-values (p ≤ 0.05 or p ≥ 0.95) may be attributable to multiple factors including violations of the assumptions underlying Carlisle’s method, strongly correlated variables, honest mistakes, poor methodology, or even fraud. The method was designed to identify issues with randomization in RCTs and appears to withstand most critiques. Although it only addresses one single dimension of a study’s potential shortcomings, that is randomization, it may nonetheless serve as a useful screening tool for a range of stakeholders, including editors or systematic reviewers who wish to flag potentially problematic trials for in-depth scrutiny prior to acceptance or inclusion. It must, however, be emphasised that the method is probabilistic in nature and cannot determine whether a specific study is definitively problematic or identify underlying causes.

## Conclusion

In this investigation of a consecutive sample of RCTs conducted in humans, we found that at least twice as many trials as expected by chance had combined p-values of p ≤ 0.05 or p ≥ 0.95, suggesting potential issues with randomization. This finding is concerning and suggests that Carlisle’s method may serve as a useful screening tool for identifying trials that warrant closer scrutiny.

## Supplementary information

Additional file 1

Additional file 2

Additional file 3

## Data Availability

The dataset supporting the main conclusion of this article is available in its Supplementary Information (Additional file 2).

## Abbreviations

Camtö: Centre for Assessment of Medical Technology in Örebro
RCT: Randomized Controlled Trial
SD: Standard Deviation
SE: Standard Error
IQR: Interquartile Ranges
AD test: Two-sample Anderson-Darling test
