# Supplementary material for: Distribution of combined p-values for baseline variables in a consecutive sample of randomized controlled trials
Source: BMC Med Res Methodol. 2026 Nov 6;26:239. doi: 10.1186/s12874-026-03082-x (PMC13744121; doi:10.1186/s12874-026-03082-x)
Supplement: Supplementary file 1 [file 12874_2026_3082_MOESM1_ESM.docx]

Supplementary material

## Example of how to apply Carlisle’s method

The first step in applying Carlisle’s method is to identify which variables and values in an RCT meet the eligibility criteria. The variables must be reported for two or more randomized groups, measured prior to the intervention, and be continuous. Categorical data cannot be used.

Baseline variables are typically found in Table 1 but can sometimes also be reported in the result tables, e.g. when laboratory measurements are compared between baseline and follow-up. The following are hypothetical examples of what might be reported in an RCT:

Example table 1 (baseline)

|  | Intervention n=31 | Control n=32 |
| --- | --- | --- |
| Age, mean (sd) | 51.7 (4.3) | 52.2 (4.7) |
| Sex, female n (%) | 22 (71.0) | 21 (65.6) |
| Weight, mean (sd) | 87.6 (5.3) | 85.4 (6.2) |

Example table 2 (results)

|  | Intervention n=29 | Control n=31 |
| --- | --- | --- |
| Hemoglobin, baseline | 156.81 (16.82) | 154.60 (13.19) |
| Hemoglobin, follow-up | 122.3 (14.3) | 143.2 (16.2) |

For Carlisle’s method, only age, weight and hemoglobin at baseline can be used. Sex is a categorical variable, and follow-up hemoglobin is measured after the intervention has started, making these variables ineligible for use with Carlisle’s method.

The extracted data are summarized as follows:

| trial | variable | number | mean | sd | decimal_mean |
| --- | --- | --- | --- | --- | --- |
| 1 | 1 | 31 | 51.7 | 4.3 | 1 |
| 1 | 1 | 32 | 52.2 | 4.7 | 1 |
| 1 | 2 | 31 | 87.6 | 5.3 | 1 |
| 1 | 2 | 32 | 85.4 | 6.2 | 1 |
| 1 | 3 | 29 | 156.81 | 16.82 | 2 |
| 1 | 3 | 31 | 154.6 | 13.19 | 2 |

- trial = An identifier (id) for each RCT (can be excluded if only one RCT will be analyzed)
- variable = An id for each baseline variable to allow the code to identify which means (sd) that measures the same thing, e.g. age for the intervention group and age for the control group
- number = Number of participants in the group, for that mean value
- mean = The mean value
- sd = The standard deviation value
- decimal_mean (optional but recommended) = The number of decimals places reported for each mean value should be extracted. Although the provided code can estimate the number of decimal places when this information is missing, it cannot detect trailing zeroes. In our example, if the number of decimal places has not been extracted, the mean 154.60 becomes 154.6, causing the number of decimal places to be underestimated as one instead of two and resulting in a loss of precision

Information on which randomized group each variable belongs to can be included, but it is not required to run the code implementing Carlisle’s method.

Once the data has been extracted, the code provided in Additional file 3 can be run in RStudio. It can then look like this:

\# Load packages

library("readxl")

library("writexl")

\# Read in the Carlisle's Method function

\# Either with source()

source("CarlislesMethodFunction.R")

\# Or add the function in the same file (copy + paste)

\# Read in the data

data <- read_excel("baseline_data.xlsx")

\# Run the function

\# Note: Check the function's arguments if your column names differ from the defaults

pval <- carlislesMethod(data = data, iterations = 100000)

pval <- as.data.frame(pval)

colnames(pval) <- c("trial","pvalue")

\# Create an excel-file with the results

write_xlsx(pval, "pval.xlsx", col_names=TRUE)

The software will recompute all p-values for the baseline variables using Student’s t-test, ANOVA and Monte Carlo simulations. Applying this to the data in the examples above yields the following p-values for each:

|  | Student’s t-test  p-values | ANOVA  p-values | Monte Carlo simulations*  p-values |
| --- | --- | --- | --- |
| Age | 0.339 | 0.339 | 0.362 |
| Weight | 0.865 | 0.864 | 0.860 |
| Hemoglobin, baseline | 0.425 | 0.428 | 0.426 |

* Due to the stochastic nature of Monte Carlo simulations, the results may differ slightly each time the simulation is run.

For each variable, the p-value that is closest to 0.5 is chosen to be used for the combined p-value. In our example, the p-value used for age would be the Monte Carlo simulation (0.362) as well as for weight (0.860), while the p-value used for hemoglobin at baseline would be the ANOVA (0.428). The calculation of the combined p-values is done with Stouffer’s method. This yields a combined p-value of 0.62. This combined p-value is neither close to 0 or 1, and does not raise any concerns about the randomization, using Carlisle’s method alone.

Note that the provided function does not return the recomputed p-values, only the combined p-value for each trial will be in the output.

Records indexed as RCTs in PubMed, February 2022 N = 1 075

Excluded n = 507 - Wrong language n = 25 - Wrong study design n = 117 - Wrong study population n = 13 - Secondary or pilot/feasibility RCT n = 200 - No baseline variable avaliable for extraction n = 152

Included n = 568

Figure S1 Flowchart

Table S1 Extreme combined p-values for included RCTs compared with Carlisle’s findings from 2017 [1], (n = 568)

| **Combined p-value** | **Observed**  **%** | **Observed**  **number of RCTs** | **Expected**  **%** | **Expected**  **number of RCTs** | **Carlisle 2017**  **%** |
| --- | --- | --- | --- | --- | --- |
| **p ≤ 0.05** | **7.9** | **45** | **5** | **28.4** | **7.1** |
| 0.05 ≥ p > 0.01 | 3.5 | 20 | 4 | 22.7 | 4.9 |
| 0.01 ≥ p > 0.001 | 2.8 | 16 | 0.9 | 5.1 | 1.5 |
| p ≤ 0.001 | 1.6 | 9 | 0.1 | 0.6 | 0.6 |
|  |  |  |  |  |  |
| **p ≥ 0.95** | **18.3** | **104** | **5** | **28.4** | **8.5** |
| 0.95 ≤ p < 0.99 | 5.8 | 33 | 4 | 22.7 | 4.9 |
| 0.99 ≤ p < 0.999 | 3.3 | 19 | 0.9 | 5.1 | 1.6 |
| p ≥ 0.999 | 9.3 | 53 | 0.1 | 0.6 | 2.0 |
|  |  |  |  |  |  |
| **p ≤ 0.05 or ≥ 0.95** | **26.2** | **149** | **10** | **56.8** | **15.6** |

Table S2 Extreme combined p-values for subgroup analyses by number of decimal places and stratification

|  |  | **Decimal**  **places ^a^** | |  | **Stratification** | |
| --- | --- | --- | --- | --- | --- | --- |
|  |  | **0-1**  n = 420 | ≥ **2**  n = 319 |  | **Stratified**  n = 131 | **Not stratified**  n = 437 |
| **Combined p-value** | **Expected %** | **Observed %** | |  | **Observed %** | |
| **p ≤ 0.05** | 5 | 5.0 | 11.6 |  | 6.1 | 8.5 |
| 0.05 ≥ p > 0.01 | 4 | 3.1 | 5.3 |  | 3.8 | 3.4 |
| 0.01 ≥ p > 0.001 | 0.9 | 1.4 | 4.4 |  | 2.3 | 3.0 |
| p ≤ 0.001 | 0.1 | 0.5 | 1.9 |  | 0.0 | 2.1 |
|  |  |  |  |  |  |  |
| **p ≥ 0.95** | 5 | 17.4 | 14.7 |  | 19.8 | 18.1 |
| 0.95 ≤ p < 0.99 | 4 | 6.4 | 3.4 |  | 6.9 | 5.5 |
| 0.99 ≤ p < 0.999 | 0.9 | 4.5 | 2.5 |  | 1.5 | 3.9 |
| p ≥ 0.999 | 0.1 | 6.4 | 8.8 |  | 11.5 | 8.7 |
|  |  |  |  |  |  |  |
| **p ≤ 0.05 or p ≥ 0.95** | 10 | 22.4 | 26.3 |  | 26.0 | 26.5 |

^a^ Some RCTs were included in both, but with different variables

Table S3 Extreme combined p-values for different assumed correlations between all variables within each RCT, by number of participants (> 100 vs ≤ 100)

|  |  | **N > 100**  n = 289 | | | |  | **N ≤ 100**  n = 279 | | | |
| --- | --- | --- | --- | --- | --- | --- | --- | --- | --- | --- |
|  |  | **Corr 0.2** | **Corr 0.4** | **Corr 0.6** | **Corr 0.8** |  | **Corr 0.2** | **Corr 0.4** | **Corr 0.6** | **Corr 0.8** |
| **Combined p-value** | **Expected %** | **Observed %** | **Observed %** | **Observed %** | **Observed %** |  | **Observed %** | **Observed %** | **Observed %** | **Observed %** |
| **p ≤ 0.05** | 5 | 3.5 | 1.4 | 0.7 | 0.3 |  | 8.6 | 5.4 | 3.6 | 1.8 |
| 0.05 ≥ p > 0.01 | 4 | 2.8 | 1.0 | 0.7 | 0.3 |  | 4.3 | 2.5 | 2.5 | 1.8 |
| 0.01 ≥ p > 0.001 | 0.9 | 0.7 | 0.3 | 0.0 | 0.0 |  | 3.2 | 2.5 | 1.1 | 0.0 |
| p ≤ 0.001 | 0.1 | 0.0 | 0.0 | 0.0 | 0.0 |  | 1.1 | 0.4 | 0.0 | 0.0 |
|  |  |  |  |  |  |  |  |  |  |  |
| **p ≥ 0.95** | 5 | 19.4 | 17.0 | 13.8 | 11.1 |  | 13.6 | 10.4 | 7.2 | 5.0 |
| 0.95 ≤ p < 0.99 | 4 | 6.6 | 5.9 | 5.2 | 4.5 |  | 5.4 | 5.0 | 3.2 | 2.9 |
| 0.99 ≤ p < 0.999 | 0.9 | 3.8 | 4.2 | 3.5 | 2.4 |  | 2.9 | 2.9 | 2.2 | 1.4 |
| p ≥ 0.999 | 0.1 | 9.0 | 6.9 | 5.2 | 4.2 |  | 5.4 | 2.5 | 1.8 | 0.7 |
|  |  |  |  |  |  |  |  |  |  |  |
| **p ≤ 0.05 or p ≥ 0.95** | 10 | 22.8 | 18.3 | 14.5 | 11.4 |  | 22.2 | 15.8 | 10.8 | 6.8 |

Table S4 Extreme combined p-values for different assumed correlations between all variables within each RCT, by number of baseline variables (> 7 vs ≤ 7)

|  |  | **> 7**  n = 272 | | | |  | **≤ 7**  n = 296 | | | |
| --- | --- | --- | --- | --- | --- | --- | --- | --- | --- | --- |
|  |  | **Corr 0.2** | **Corr 0.4** | **Corr 0.6** | **Corr 0.8** |  | **Corr 0.2** | **Corr 0.4** | **Corr 0.6** | **Corr 0.8** |
| **Combined p-value** | **Expected %** | **Observed %** | **Observed %** | **Observed %** | **Observed %** |  | **Observed %** | **Observed %** | **Observed %** | **Observed %** |
| **p ≤ 0.05** | 5 | 8.1 | 3.3 | 1.5 | 0.0 |  | 4.1 | 3.4 | 2.7 | 2.0 |
| 0.05 ≥ p > 0.01 | 4 | 4.8 | 1.8 | 1.5 | 0.0 |  | 2.4 | 1.7 | 1.7 | 2.0 |
| 0.01 ≥ p > 0.001 | 0.9 | 2.6 | 1.5 | 0.0 | 0.0 |  | 1.4 | 1.4 | 1.0 | 0.0 |
| p ≤ 0.001 | 0.1 | 0.7 | 0.0 | 0.0 | 0.0 |  | 0.3 | 0.3 | 0.0 | 0.0 |
|  |  |  |  |  |  |  |  |  |  |  |
| **p ≥ 0.95** | 5 | 19.5 | 14.0 | 8.8 | 5.9 |  | 14.2 | 13.5 | 12.2 | 10.1 |
| 0.95 ≤ p < 0.99 | 4 | 6.6 | 5.9 | 3.3 | 1.8 |  | 5.7 | 5.1 | 5.1 | 5.4 |
| 0.99 ≤ p < 0.999 | 0.9 | 4.4 | 2.6 | 1.5 | 2.2 |  | 2.4 | 4.4 | 4.1 | 1.7 |
| p ≥ 0.999 | 0.1 | 8.5 | 5.5 | 4.0 | 1.8 |  | 6.1 | 4.1 | 3.0 | 3.0 |
|  |  |  |  |  |  |  |  |  |  |  |
| **p ≤ 0.05 or p ≥ 0.95** | 10 | 27.6 | 17.3 | 10.3 | 5.9 |  | 18.2 | 16.9 | 14.9 | 12.2 |

Table S5 Extreme combined p-values for different assumed correlations between all variables within each RCT, by number of decimal places (0-1 vs ≥ 2)

|  |  | **0-1 ^a^**  n = 420 | | | |  | ≥ **2 ^a^**  n = 319 | | | |
| --- | --- | --- | --- | --- | --- | --- | --- | --- | --- | --- |
|  |  | **Corr 0.2** | **Corr 0.4** | **Corr 0.6** | **Corr 0.8** |  | **Corr 0.2** | **Corr 0.4** | **Corr 0.6** | **Corr 0.8** |
| **Combined p-value** | **Expected %** | **Observed %** | **Observed %** | **Observed %** | **Observed %** |  | **Observed %** | **Observed %** | **Observed %** | **Observed %** |
| **p ≤ 0.05** | 5 | 3.8 | 1.7 | 0.7 | 0.2 |  | 10.0 | 7.5 | 5.6 | 3.4 |
| 0.05 ≥ p > 0.01 | 4 | 3.3 | 1.4 | 0.7 | 0.2 |  | 5.6 | 4.7 | 4.1 | 3.1 |
| 0.01 ≥ p > 0.001 | 0.9 | 0.2 | 0.2 | 0.0 | 0.0 |  | 3.8 | 2.5 | 1.6 | 0.3 |
| p ≤ 0.001 | 0.1 | 0.2 | 0.0 | 0.0 | 0.0 |  | 0.6 | 0.3 | 0.0 | 0.0 |
|  |  |  |  |  |  |  |  |  |  |  |
| **p ≥ 0.95** | 5 | 15.5 | 12.4 | 8.8 | 7.6 |  | 14.1 | 11.6 | 11.0 | 8.8 |
| 0.95 ≤ p < 0.99 | 4 | 6.4 | 6.0 | 3.1 | 2.9 |  | 4.1 | 1.9 | 4.4 | 5.0 |
| 0.99 ≤ p < 0.999 | 0.9 | 3.3 | 1.9 | 2.1 | 1.7 |  | 2.2 | 4.7 | 3.1 | 1.3 |
| p ≥ 0.999 | 0.1 | 5.7 | 4.5 | 3.6 | 3.1 |  | 7.8 | 5.0 | 3.4 | 2.5 |
|  |  |  |  |  |  |  |  |  |  |  |
| **p ≤ 0.05 or p ≥ 0.95** | 10 | 19.3 | 14.0 | 9.5 | 7.9 |  | 24.1 | 19.1 | 16.6 | 12.2 |

^a^ Some RCTs were included in both, but with different variables

Table S6 Extreme combined p-values for different assumed correlations between all variables within each RCT, by stratification status

|  |  | **Stratified**  n = 131 | | | |  | **Not stratified**  n = 437 | | | |
| --- | --- | --- | --- | --- | --- | --- | --- | --- | --- | --- |
|  |  | **Corr 0.2** | **Corr 0.4** | **Corr 0.6** | **Corr 0.8** |  | **Corr 0.2** | **Corr 0.4** | **Corr 0.6** | **Corr 0.8** |
| **Combined p-value** | **Expected %** | **Observed %** | **Observed %** | **Observed %** | **Observed %** |  | **Observed %** | **Observed %** | **Observed %** | **Observed %** |
| **p ≤ 0.05** | 5 | 3.8 | 1.5 | 0.8 | 0.0 |  | 6.6 | 3.9 | 2.5 | 1.4 |
| 0.05 ≥ p > 0.01 | 4 | 3.8 | 1.5 | 0.8 | 0.0 |  | 3.2 | 1.8 | 1.8 | 1.4 |
| 0.01 ≥ p > 0.001 | 0.9 | 0.0 | 0.0 | 0.0 | 0.0 |  | 2.7 | 1.8 | 0.7 | 0.0 |
| p ≤ 0.001 | 0.1 | 0.0 | 0.0 | 0.0 | 0.0 |  | 0.7 | 0.2 | 0.0 | 0.0 |
|  |  |  |  |  |  |  |  |  |  |  |
| **p ≥ 0.95** | 5 | 17.6 | 14.5 | 11.5 | 7.6 |  | 16.2 | 13.3 | 10.1 | 8.2 |
| 0.95 ≤ p < 0.99 | 4 | 6.1 | 6.1 | 4.6 | 2.3 |  | 5.9 | 5.0 | 3.9 | 4.1 |
| 0.99 ≤ p < 0.999 | 0.9 | 2.3 | 3.1 | 2.3 | 2.3 |  | 3.7 | 3.7 | 3.0 | 1.8 |
| p ≥ 0.999 | 0.1 | 9.2 | 5.3 | 4.6 | 3.1 |  | 6.6 | 4.6 | 3.2 | 2.3 |
|  |  |  |  |  |  |  |  |  |  |  |
| **p ≤ 0.05 or p ≥ 0.95** | 10 | 21.4 | 16.0 | 12.2 | 7.6 |  | 22.9 | 17.2 | 12.6 | 9.6 |

Table S7 Extreme combined p-values for the sensitivity analyses using Fisher’s method instead of Stouffer’s method, Monte Carlo simulations only, and reported measures of central tendency

|  |  |  | **Fisher’s method instead of Stouffer’s method** |  | **Only Monte Carlo simulations for recalculated p-values** |  | **Central**  **tendency ^a^** | |
| --- | --- | --- | --- | --- | --- | --- | --- | --- |
|  |  |  | n = 568 |  | n = 568 |  | **Medians**  n = 177 | **Means**  n = 498 |
| **Combined p-value** | **Expected %** |  | **Observed %** |  | **Observed %** |  | **Observed %** | |
| **p ≤ 0.05** | 5 |  | 7.6 |  | 8.5 |  | 4.5 | 8.2 |
| 0.05 ≥ p > 0.01 | 4 |  | 4.2 |  | 4 |  | 4.0 | 3.6 |
| 0.01 ≥ p > 0.001 | 0.9 |  | 2.3 |  | 2.8 |  | 0.6 | 2.8 |
| p ≤ 0.001 | 0.1 |  | 1.1 |  | 1.6 |  | 0.0 | 1.8 |
|  |  |  |  |  |  |  |  |  |
| **p ≥ 0.95** | 5 |  | 14.1 |  | 19.5 |  | 24.3 | 15.7 |
| 0.95 ≤ p < 0.99 | 4 |  | 6.7 |  | 5.8 |  | 8.5 | 4.6 |
| 0.99 ≤ p < 0.999 | 0.9 |  | 3.2 |  | 3.3 |  | 6.2 | 3.4 |
| p ≥ 0.999 | 0.1 |  | 4.2 |  | 10.4 |  | 9.6 | 7.6 |
|  |  |  |  |  |  |  |  |  |
| **p ≤ 0.05 or p ≥ 0.95** | 10 |  | 21.7 |  | 28.0 |  | 28.8 | 23.9 |

^a^ Some RCTs were included in both, but with different variables

Figure S2 Distribution of combined p-values of RCTs with 100 or less participants (N ≤ 100), n=279, showed by a) a histogram and b) a Q-Q plot with a 99% confidence interval for the expected uniform distribution.

Figure S3 Distribution of combined p-values of RCTs with more than 100 participants (N > 100), n=289, showed by a) a histogram and b) a Q-Q plot with a 99% confidence interval for the expected uniform distribution.

Figure S4 Distribution of combined p-values of RCTs with ≤ 7 variables, n=296, showed by a) a histogram and b) a Q-Q plot with a 99% confidence interval for the expected uniform distribution.

Figure S5 Distribution of combined p-values of RCTs with > 7 variables, n=272, showed by a) a histogram and b) a Q-Q plot with a 99% confidence interval for the expected uniform distribution.

**Reference**

1.	Carlisle JB. Data fabrication and other reasons for non-random sampling in 5087 randomised, controlled trials in anaesthetic and general medical journals. Anaesthesia. 2017;72:944-52. doi:10.1111/anae.13938.
