# Supplementary material for: Distribution of combined p-values for baseline variables in a consecutive sample of randomized controlled trials
Source: BMC Med Res Methodol. 2026 Nov 6;26:239. doi: 10.1186/s12874-026-03082-x (PMC13744121; doi:10.1186/s12874-026-03082-x)
Supplement: Supplementary file 2 [file 12874_2026_3082_MOESM3_ESM.docx]

\# Function for Carlisle's method

\### Code is based on:

\# Carlisle's comment on the blog post

\# “Exploring John Carlisle's "bombshell" article about fabricated data in RCTs”

\# In Nick Brown's blog, 2017 June 8

\# http://steamtraen.blogspot.com/2017/06/exploring-john-carlisles-bombshell.html?showComment=1496952386470#c6997813275565739633

\# Accessed 2024 July 16

\# Carlisle JB, Loadsman JA

\# Evidence for non-random sampling in randomised, controlled trials by Yuhji Saitoh

\# Anaesthesia. 2017;72:17-27.

\# doi:10.1111/anae.13650

\# Carlisle JB, Dexter F, Pandit JJ, Shafer SL, Yentis SM

\# Calculating the probability of random sampling for continuous variables in submitted or published randomised controlled trials

\# Anaesthesia. 2015;70:848-58.

\# doi:10.1111/anae.13126

\# Example of how it's used:

\# # Load packages

\# library("readxl")

\# library("writexl")

\# # Read in the Carlisle's Method function

\# # Either with source()

\# source("CarlislesMethodFunction.R")

\# # Or add the function here

\# # Read in the data

\# data <- read_excel("baseline_data_for_568_RCTs.xlsx")

\# # Run the function

\# # Note: Check the function's arguments if your column names differ from the defaults

\# pval <- carlislesMethod(data = data, iterations = 100000)

\# pval <- as.data.frame(pval)

\# colnames(pval) <- c("trial","pvalue")

\# # Create an excel-file with the results

\# write_xlsx(pval, "pval.xlsx", col_names=TRUE)

library("BSDA")

library("CarletonStats")

library("dplyr")

library("MBESS")

library("poolr")

\# Function for Carlisle's method

\# Arguments:

\# data = dataset with all the extracted baseline variables

\# iterations = number of iterations done with the Monte Carlo simulation

\# Lower "iterations" if the code takes too long to complete

\# id = id number/text if the data includes more than one trial

\# variable = identification of which means (sd) are connected in a trial

\# number = number of participants in a group

\# mean & sd = the mean and standard deviation of a baseline variable

\# decimal_mean = number of decimals for the mean

carlislesMethod <- function(data,

iterations = 100000,

id = "trial",

variable = "variable",

number = "number",

mean = "mean",

sd = "sd",

decimal_mean = "decimal_mean") {

\# If a needed argument is not given, stop and give an error message

if (!(variable %in% colnames(data))) {

stop("Error: The 'variable' argument is required.")

}

if (!(number %in% colnames(data))) {

stop("Error: The 'number' argument is required.")

}

if (!(mean %in% colnames(data))) {

stop("Error: The 'mean' argument is required.")

}

if (!(sd %in% colnames(data))) {

stop("Error: The 'sd' argument is required.")

}

\# If decimal argument is missing, count decimals

if (!(decimal_mean %in% colnames(data))) {

data[[decimal_mean]] <- sapply(data[[mean]], function(x) {

if ((x %% 1) != 0) {

nchar(strsplit(sub('0+$', '', as.character(x)),

".", fixed = TRUE)[[1]][[2]])

} else {

return(0)

}

})

}

\# Check if the id argument is provided

\# or if there are multiple unique trial values

if (id %in% colnames(data)) {

\# Create id numbers for each trial

data[["trialid"]] <- as.integer(factor(data[[id]],

levels = unique(data[[id]])))

} else {

\# If no id is provided, it is assumed that there is only one unique trial

data[["trialid"]] <- 1

}

\# Order the data

data <- data[order(data[["trialid"]], data[[variable]]), ]

\# Total number of trials

Maxtrial <- max(data[["trialid"]])

\# Make sure each variable is numbered 1,2,3... and doesn't jump numbers

data[["variable_trial"]] <- NA

for (i in 1:Maxtrial) {

v <- unique(data[data[["trialid"]] == i, ][[variable]])

a <- 1

for (j in 1:sum(data[["trialid"]] == i)) {

if (data[data[["trialid"]] == i, ][[variable]][j] == v[a]) {

data[data[["trialid"]] == i, ][["variable_trial"]][j] <- a

} else {

data[data[["trialid"]] == i, ][["variable_trial"]][j] <- a + 1

a <- a + 1

}

}

}

\# Number of variables for each trial

MaxC1 <- NA

for (i in 1:Maxtrial) {

C1 <- which(data[["trialid"]] == i)

Maxmeasure <- length(unique(data[[variable]][C1]))

MaxC1[i] <- Maxmeasure

}

\# Generate mid p-values for each trial with Monte Carlo simulation

Ptrial <- matrix(data = NA, nrow = Maxtrial, ncol = max(MaxC1, na.rm = T))

for (i in 1:Maxtrial) {

PPmean <- NA

\# Maximum number of variables that any trial in the data has

length(PPmean) <- max(MaxC1, na.rm = T)

\# Generate mid p-values for each variable in trial i

for (j in 1:MaxC1[i]) {

Row <- data[["trialid"]] == i & data[["variable_trial"]] == j

Length <- length(which(Row))

Lines <- which(Row)

Sample <- Value <- Participants <- Standard <- rep(1:Length)

DiffSample <- rep(1:iterations)

\# Extract values for each row of interest (i.e. variable j in trial i)

for (k in 1:Length) {

\# Total for line k (number in group * mean)

Value[k] <- data[[number]][Lines[k]] * data[[mean]][Lines[k]]

\# Number in group at line k

Participants[k] <- data[[number]][Lines[k]]

\# If number in group is bigger than 300, return the sd given in article,

\# if not, then give a corrected version of the sd

SSDD <- ifelse(data[[number]][Lines[k]] > 300,

data[[sd]][Lines[k]],

s.u(data[[sd]][Lines[k]], data[[number]][Lines[k]]))

\# Total of variance for line k (number in group * sd^2)

Standard[k] <- data[[number]][Lines[k]] * SSDD^2

}

\# Various calculations for mean, sd and sem for variable j

\# Combined mean

Meanmean <- sum(Value) / sum(Participants)

\# Combined variance

Meanvar <- sum(Standard) / sum(Participants)

\# Combined sd

Meansd <- Meanvar^0.5

\# Mean number of people per group

MeanNo <- sum(Participants) / Length

\# Sum of the squared differences in the means (Box 5 in Carlisle, 2015)

Diff <- sum((data[[mean]][Row] - Meanmean)^2)

\# Standard error of the sample mean

SEMsample <- Meansd / sqrt(MeanNo)

\# Simulate samples

for (k in 1:iterations) {

\# Simulated mean

meansim <- rnorm(1, mean = Meanmean, sd = SEMsample)

\# Generate a sample for each variable that's rounded to the same

\# number of decimals as the observed mean in the article

for (l in 1:Length) {

Sample[l] <- round(sum(rnorm(data[[number]][Lines[l]],

mean = meansim,

sd = Meansd)

/ data[[number]][Lines[l]]),

data[[decimal_mean]][Lines[l]])

}

\# Estimation of the population mean (Box 1 step 1 in Carlisle, 2015)

MeanSample <- sum(Sample * Participants) / sum(Participants)

\# Sum of the squared differences in the means (Box 5 in Carlisle, 2015)

DiffSample[k] <- sum((Sample - MeanSample)^2)

}

\# Calculate mid p-values

\# Number of simulated mean differences smaller than

\# observed difference divided by number of simulations

PP1 <- sum(DiffSample < Diff) / iterations

\# Number of simulated mean differences smaller than or equal to

\# observed difference divided by number of simulations

PP2 <- sum(DiffSample <= Diff) / iterations

\# Mid p-value for variable j

PPmean[j] <- (PP1 + PP2) / 2

\# Handle 0 and 1 by making it either slightly bigger or slightly smaller

if (PPmean[j] == 0) {

PPmean[j] <- 1e-16

} else if (PPmean[j] == 1) {

PPmean[j] <- 1 - 1e-16

}

}

\# Store mid p-values for trial i in row i

Ptrial[i, ] <- PPmean

}

\# Generate ANOVA p-values for each variable in each trial

ANOVAtable <- NA

ANOVAP <- matrix(data = NA, nrow = Maxtrial, ncol = max(MaxC1, na.rm = T))

for (i in 1:Maxtrial) {

for (j in 1:MaxC1[i]) {

Row <- data[["trialid"]] == i & data[["variable_trial"]] == j

Lines <- which(Row)

ANOVAtable <- invisible(anovaSummarized(data[[number]][Lines],

data[[mean]][Lines],

data[[sd]][Lines]))

\# Store the inverse of the p-value from the ANOVA F test

ANOVAP[i,j] <- 1 - (as.numeric(ANOVAtable[8]))

}

}

\# Generate t-test p-values for each variable in each trial

TTESTP <- matrix(data = NA, nrow = Maxtrial, ncol = max(MaxC1, na.rm = T))

for (i in 1:Maxtrial) {

for (j in 1:MaxC1[i]) {

Row <- data[["trialid"]] == i & data[["variable_trial"]] == j

Lines <- which(Row)

\# If we're looking at more than 2 rows, return NA,

\# else do a two-sample t-test between the two lines and

\# return the inverted p-value

ifelse(length(Lines) > 2, TTESTP[i,j] <- NA,

TTESTP[i,j] <- 1 - (tsum.test(data[[mean]][Lines[1]],

data[[sd]][Lines[1]],

data[[number]][Lines[1]],

data[[mean]][Lines[2]],

data[[sd]][Lines[2]],

data[[number]][Lines[2]])[["p.value"]]))

}

}

\# Find the p-value closest to 0.5 for each variable in each trial

MinP <- matrix(data = NA, nrow = Maxtrial, ncol = max(MaxC1, na.rm = T))

for (i in 1:Maxtrial) {

for (j in 1:MaxC1[i]) {

\# Vector with the p-values from Monte Carlo, ANOVA and t test

\# for variable j in trial i

choice <- c(Ptrial[i,j], ANOVAP[i,j], TTESTP[i,j])

\# Monte Carlo diff from 0.5 squared (the closer to 0.5, the smaller)

MCP <- (Ptrial[i,j] - 0.5)^2

\# ANOVA diff from 0.5 squared (the closer to 0.5, the smaller)

ANP <- (ANOVAP[i,j] - 0.5)^2

\# t test diff from 0.5 squared (the closer to 0.5, the smaller)

TTP <- (TTESTP[i,j] - 0.5)^2

\# Picks the closest p-value to 0.5

near0.5 <- which.min(c(MCP, ANP, TTP))

\# Save the p-value that is the closest to 0.5

MinP[i,j] <- choice[near0.5]

}

}

MinP <- as.data.frame(MinP)

Pvartrial <- matrix(data = NA, nrow = Maxtrial, ncol = 2)

if (id %in% colnames(data)) {

trials <- unique(data[[id]])

} else {

trials <- 1

}

\# Which trials that only have 1 variable

measures1 <- which(MaxC1 == 1)

\# Which trials that have more than 1 variable

measures2 <- which(MaxC1 > 1)

\# If a trial only has 1 variable, pick the p-value that is closest to 0.5

for (i in measures1) {

Pvartrial[i,1] <- trials[i]

Pvartrial[i,2] <- MinP[i,1]

}

\# If a trial has more than 1 variable,

\# combine the p-values with Stouffer's method

for (i in measures2) {

z <- which(!is.na(MinP[i, ]))

p <- as.numeric(MinP[i,z])

pcomb <- stouffer(p)

Pvartrial[i,1] <- trials[i]

Pvartrial[i,2] <- pcomb$p

}

return(Pvartrial)

}
